# Lyon bracing in adolescent females with thoracic idiopathic scoliosis: a prospective study based on SRS and SOSORT criteria

**DOI:** 10.1186/s12891-015-0782-0

**Published:** 2015-10-24

**Authors:** Angelo G. Aulisa, Vincenzo Guzzanti, Francesco Falciglia, Marco Giordano, Emanuele Marzetti, Lorenzo Aulisa

**Affiliations:** U.O.C. of Orthopedics and Traumatology, Children’s Hospital Bambino Gesù, Institute of Scientific Research, P.zza S. Onofrio 4, Rome, 00165 Italy; University of Cassino, Cassino, FR 03043 Italy; Department of Orthopedics, University Hospital “Agostino Gemelli”, Catholic University of the Sacred Heart School of Medicine, Rome, 00168 Italy

**Keywords:** Adolescent idiopathic scoliosis, Scoliosis Research Society Criteria (SRS), Lyon brace, SOSORT guidelines, conservative treatment

## Abstract

**Background:**

The Lyon brace is commonly prescribed in many European countries to patients with thoracic curves and is based on the three-point pressure system.

The purpose of this study was to evaluate the efficacy of Lyon bracing for the conservative treatment of adolescent females with idiopathic thoracic curves in a case series selected on the basis of the Scoliosis Research Society (SRS) Committee on Bracing and Nonoperative Management Standardization Criteria and followed the guidelines on management of idiopathic scoliosis with corrective braces, proposed by the International Society on Scoliosis Orthopaedic and Rehabilitation Treatment (SOSORT).

**Methods:**

Prospective study based on an ongoing database.

From 1297 patients treated for idiopathic scoliosis between 1995 and 2014 fulfill the inclusion criteria 102 patients treated with Lyon brace. Of these, 69 patients had a definite outcome, 17 have abandoned treatment e 16 are still in treatment. The 104 patients were adolescent females with curvatures in the thoracic spine and a pre-treatment Risser score ranging from 0 to 2. All patients were prescribed with full-time Lyon bracing. The minimum duration of follow-up was 24 months after the end of weaning (mean: 41.64 ± 31.45 months). Anteroposterior radiographs were used to estimate the curve magnitude (C_M_) at 5 time points: beginning of treatment (t1), one year after the beginning of treatment (t2), intermediate time between t1 and t4 (t3), end of weaning (t4), 2-year minimum follow-up from t4 (t5). Three outcomes were distinguished: curve correction, curve stabilization and curve progression.

**Results:**

The results from our study showed that of the 69 patients with a definite outcome the C_M_ mean value was 31.51° ± 4.34 SD at t1 and 20° ± 7.6 SD at t5. Curve correction was accomplished in 85.5 % of patients, curve stabilization was obtained in 13 % of patients and curve progression was evident in only 1.5 %. None of the patients were recommended surgery post-bracing. Of 17 patients who abandoned the treatment, at the time of abandonment (14.4 age) have achieved curve correction in 13 cases (77 %), stabilization in 53 cases (18 %) and progression in 1 case (5 %).

**Conclusion:**

The Lyon brace, through its biomechanical action on vertebral modeling, is highly effective in correcting thoracic curves in particularly when the SOSORT guidelines were adopted in addition to the SRS criteria.

## Background

Major thoracic curves are the most common scoliotic curve type. These curves account for approximately 30 % of cases of moderate Adolescent Idiopathic Scoliosis (AIS) in both sexes, and for 35 % and 60 % of severe AIS in males and females, respectively [1].

The Lyon brace, devised by Stagnara in 1947, is commonly prescribed in many European countries to patients with thoracic curves and is based on the three-point pressure system [2]. The Lyon brace applies derotational forces to the spine; in the frontal plane, the action is performed according to the three point system. Thoracic derotation is obtained through a pad applied at the internal side of the rib hump and an anterior chondrocostal concave counter-pad. At the lumbar level, the push is realized on a convex transverse. In the sagittal plane, the lumbar lordosis is accentuated in order to increase the kyphosis of the thoracic region by sagittal bending of the bars. In a recent study, the minimal curve indication for Lyon bracing was set at ≥ 20° during the phase of accelerated growth for 11 to 13-year-old patients, and 30° during the phase of slow pubertal growth, or >40° when surgery is refused by the patient [3].

The Lyon brace is particularly suitable to patients during fast pubertal growth, while other orthoses (e.g., Milwaukee brace) are more indicated for pre-pubertal juvenile patients [3].

Although Lyon bracing is generally considered an effective means for the conservative management of AIS, to our knowledge, no studies have specifically evaluated its efficacy in the context of the recently published Scoliosis Research Society (SRS) Committee on Bracing and Nonoperative Management Standardization Criteria [4, 5].

According to the SRS guidelines, only scoliotic patients with the following characteristics should be included in clinical trials evaluating the effects of conservative treatment: age 10 years or older when bracing is started; Risser score 0–2; primary curve angles 25–40° Cobb; no prior treatment for scoliosis; if female, either premenarchal or less than 1 year postmenarchal [5].

The present study was therefore undertaken to determine the efficacy of Lyon bracing in the correction of thoracic curves in adolescent females, according to the SRS recommendations [5]. The study also followed the guidelines on standard of management of idiopathic scoliosis with corrective braces in everyday clinics and in clinical research, proposed by the International Society on Scoliosis Orthopaedic and Rehabilitation Treatment (SOSORT) [6].

## Methods

### Study design and participants

This is a prospective study based on ongoing database including 1297 patients treated for idiopathic scoliosis between 1995 and 2014. It was conducted in respect to the Helsinki Declaration, and all the participants (parents) signed and informed consent to allow the use of clinical data for research purpose. The study has been approved by the Ethics Committee of the Ospedale Pediatrico Bambino Gesù in Rome, Italy. Of over 300 scoliotic patients treated with the Lyon brace, 148 young girls met the SRS criteria. The remaining cases were excluded due to incomplete fulfilling of SOSORT management criteria, such as double scoliotic curves or infantile and juvenile scoliosis. Among the 148 patients, forty-six cases were excluded because they presented with thoracolumbar curves. Therefore, 102 adolescent females (mean age 11.62 ± 1.1 years) with thoracic curves met the inclusion criteria of the SRS Committee and were therefore included in the analyses. All patients presented with a single major thoracic curve, ranging in magnitude between 25 and 40° Cobb. The age at the beginning of treatment was 10–12 years, while the Risser score was comprised between 0 and 2.

One-hundred two patients met the inclusion criteria. Of these, 69 patients had definite outcome, 17 abandoned the treatment, and 16 are currently under treatment.

### Bracing protocol

All patients were prescribed with full-time (maximum 22 hours daily, minimum 18 hours daily) Lyon bracing. Curve progression was assessed on two consecutive X-rays taken at 6-month intervals.

Progression was defined as an increase > 5° in both curve magnitude (Cobb’s method) and apical torsion (Perdriolle’s method) [7, 8]. Weaning was started when ring-apophysis fusion was seen to begin on a latero-lateral (LL) view X-ray, and consisted of 2–4 hours bracing reduction at 2-month intervals for a total of 8–10 months of weaning. The curve response to part-time bracing was monitored on an anterior-posterior (AP) view standing radiograph after the patient had been without brace for 5 hours. This interval has been chosen based on our experience that it is sufficient to minimize the interference of bracing on the imaging outcome. Out-of-brace hours were not reduced, and in some cases increased, if the curve was not stable. Treatment was concluded when the ring-apophysis fusion was complete on a LL X-ray [9]. A minimum follow-up of 24 months after the end of treatment was performed. Daily hours of bracing were individualized based on the patient clinical needs and acceptance. Compliance to treatment was established via in-person interviews. In order to maximize treatment adherence, patients were always followed by the same physician. Frequently checks were also necessary to maximize the efficacy of bracing over time.

Controls were performed every two months until Risser 3, in which the growth and the morphological and structural changes of the trunk occur more quickly, and every three months thereafter. No physiotherapy program was prescribed.

### Endpoints

X-rays were taken at conventional times: beginning of treatment (t1), one year after the beginning of treatment (t2), intermediate time between t1 and t4 (t3), end of weaning (t4), 2-year minimum follow up from t4 (t5). The X-rays in t2 and t3 are in brace, they are taken while in the brace, as they are useful to check the corrective action of the brace. For each patient, AP and LL view standing X-rays of the whole spine were performed. All radiographs were taken at a 2-meter distance on a 36x91 cm film. The AP view was used to determine the patient’s skeletal age (Risser’s sign), the curve magnitude (C_M_, Cobb’s method) and the torsion of the apical vertebra (T_A_: Perdriolle’s method). Measurements were obtained by two independent observers. End-vertebrae were pre-selected to minimize the interobserver error [7]. The interobserver concordance as assessed by the Cohen K statistic was high (0.82).

### Statistical analysis

Normality of data was ascertained via the Kolmogorov-Smirnov test. Changes in C_M_, and T_A_ from t1 through t5 were assessed via one-way analysis of variance (ANOVA) for repeated measures, followed by Bonferroni’s post-hoc test when needed. Mean differences between time-points and 95 % confidence intervals (CIs) were calculated. Correlations between changes of C_M_ between t1 and t5 (C_M_ t5-t1), and T_A_ between t1 and t5 (T_A_ t5-t1) were determined via the Pearson’s test. The same test was used to explore correlations between changes in radiographic parameters (C_M_ t5-t1 and T_A_ t5-t1) and patient’s age at t1. Finally, results were analyzed in relation to C_M_ t5-t1 at follow-up, assuming that C_M_ t5-t1 was not within the Cobb’s method ± 5 range error [7].

Three possible outcomes were distinguished: curve correction (C_M_ t5-t1 ≤ −5° Cobb), curve stabilization (C_M_ t5-t1 > −5 and < 5° Cobb) and curve progression (C_M_ t5-t1 ≥ 5° Cobb). All analyses were performed according to the intention-to-treat principle as recommended by the SRS committee. All tests were two-sided, with significance set at *p* <0.05. Results are presented as mean ± standard deviation (SD).

## Results

### Analyses of patients with a definite outcome

**Fig. 1:**
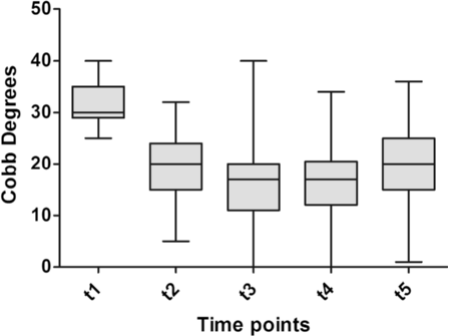
Changes in curve magnitude in Cobb degrees from the beginning of treatment (t1) to 2-year minimum follow-up from end of weaning (t5). Each box depicts the interquartile range, with the median indicated by the the black center line. Error bars show the data distribution, with the whiskers corresponding to the minimum and maximum values

**Fig. 2:**
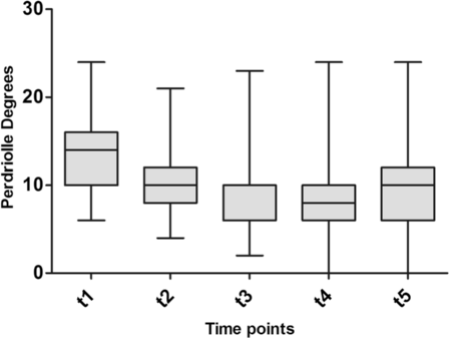
Changes in apical torsion inPerdriolle degrees from the beginning of treatment (t1) to 2-year minimum follow-up from end of weaning (t5). Each box depicts the interquartile range, with the median indicated by the the black center line. Error bars show the data distribution, with the whiskers corresponding to the minimum and maximum values

**Fig. 3:**
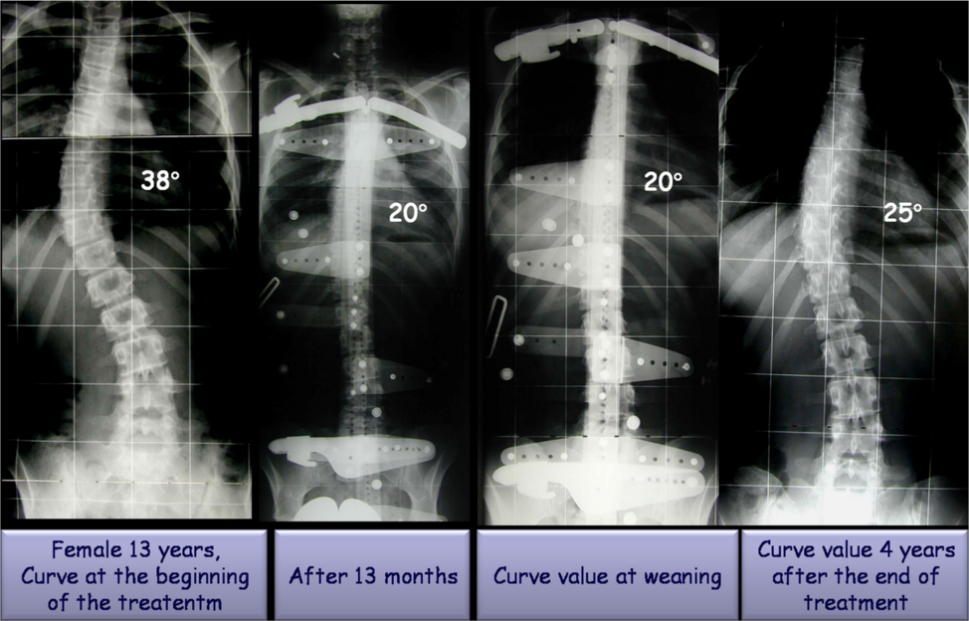
The figure shows a case with a curve value of 38° Cobb at beginning of treatment and 25° Cobb at 4 years of follow-up

**Fig. 4:**
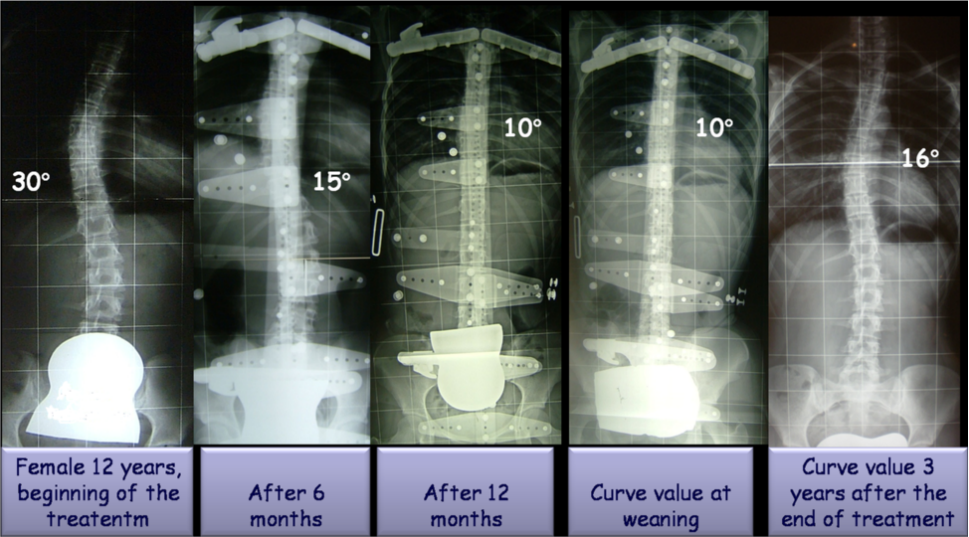
The figure shows a case with a curve value of 30° Cobb at beginning of treatment and 16° Cobb at 3 years of follow-up

Demographic and radiological characteristics of the study sample across time-points are summarized in Table 1. All patients were followed until t5. The mean duration of treatment was 62.9 ± 15.03 months (range: 21–98; median: 60), whereas the average length of follow-up was 41.64 ± 31.45 months after the end of weaning (range: 24–158; median: 34). The Cobb degrees mean value was 31.51 ± 4.34 SD at beginning of treatment (t1) and 20 ± 7.6 SD at end of follow up (t5). The Perdriolle degrees mean value was 13.09 ± 3.56 SD at beginning of treatment (t1) and 9.21 ± 4.5 SD at end of follow up (t5). Significant differences were determined for both C_M_ and T_A_ across t1-t5 (Tables 2–3; Figs. 1–2). In addition, the following correlations were calculated: C_M_ t5-t1 and T_A_ t5-t1 (Pearson’s *r* = −0.1559, *p* = 0.6285); C_M_ t5-t1 and patient’s age at t1 (Pearson’s *r* = 0.2949, *p* = 0.0146); and T_A_ t5-t1 and patient’s age at t1 (Pearson’s *r* = −0.3349, *p* = 0.0945). Fifty-nine patients out 69 (85.5 %) obtained a curve correction (mean C_M_ t5-t1: −13.08 ± 5.5° Cobb), whilst a curve stabilization was achieved in nine patients (13 %) (mean C_M_ t5-t1: −1.11 ± 2.02° Cobb). Only one patient presented a curve progression (1.5 %) (mean C_M_ t5-t1: 5 ± 0° Cobb) after brace treatment and None of the 69 patients were recommended surgery post-bracing (Figs. 3–4).The overall compliance to treatment was satisfactory, with 84 % of patients reporting complete adherence to the prescription.

**Table 1:** Demographic and radiological characteristics of the study sample

	Beginning of treatment (t_1_)	Beginning of weaning	End of treatment (t_4_)	End of follow-up (t_5_)
Age (years)	12.3 ± 1.3	17.4 ± 1.5	18.5 ± 1.5	41.6 ± 31.4
Cobb degrees	31.5 ± 4.3	16.6 ± 9.0	16.3 ± 9.6	20 ± 7.6
Perdriolle degrees	13.1 ± 3.5	8.6 ± 4.5	8.1 ± 4.1	9.2 ± 4.5

**Table 2:** Differences in C_M_ across t1-t5 as determined by one-way ANOVA with Bonferroni’s post test

Comparison	Mean C_M_ difference	95 % CI	*P* value
t_1_ vs. t_2_	12.4	6.7 – 18.1	<0.0001
t_1_ vs. t_3_	14.9	9.2 – 20.6	<0.0001
t_1_ vs. t_4_	15.1	9.4 – 20.8	<0.0001
t_1_ vs. t_5_	11.5	5.8 – 17.2	<0.0001
t_2_ vs. t_3_	2.5	−3.2 – 8.1	>0.05
t_2_ vs. t_4_	2.7	−3.0 – 8.2	>0.05
t_2_ vs. t_5_	−0.9	−6.6 – 4.8	>0.05
t_3_ vs. t_4_	0.2	−5.4 – 5.9	>0.05
t_3_ vs. t_5_	−3.4	−9.1 – 2.3	>0.05
t_4_ vs. t_5_	−3.6	−9.3 – 2.1	>0.05

ANOVA indicates analysis of variance; CI, confidence interval; t1, beginning of treatment; t2, 1 year after the beginning of treatment; t3, intermediate time between t1 and t4; t4 , end of weaning; t5, 2-year minimum follow-up from t4

**Table 3:** Differences in T_A_ across t1-t5 as determined by one-way ANOVA with Bonferroni’s post test

Comparison	Mean T_A_ difference	95 % CI	*P* value
t_1_ vs. t_2_	2.8	−2.8 – 8.5	<0.0001
t_1_ vs. t_3_	4.5	−1.2 – 10.2	<0.0001
t_1_ vs. t_4_	4.9	−0.7 – 10.7	<0.0001
t_1_ vs. t_5_	3.8	−1.8 – 9.5	<0.0001
t_2_ vs. t_3_	1.6	−4.0 – 7.3	>0.05
t_2_ vs. t_4_	2.1	−3.6 – 7.8	<0.0001
t_2_ vs. t_5_	0.9	−4.7 – 6.6	>0.05
t_3_ vs. t_4_	0.4	−5.2 – 6.2	>0.05
t_3_ vs. t_5_	−0.6	−6.3 – 5.1	>0.05
t_4_ vs. t_5_	−1.1	−6.8 – 4.5	>0.05

ANOVA indicates analysis of variance; CI, confidence interval; t1, beginning of treatment; t2, 1 year after the beginning of treatment; t3, intermediate time between t1 and t4; t4, end of weaning; t5, 2-year minimum follow-up from t4

### Analyses of patients who abandoned the treatment

Seventeen patients abandoned the treatment, mean age: 11.5 years at t1 and 14.4 years at the time of discontinuation.

CM was 30.2 ± 5.59° Cobb at t1 and 18.7 ± 8.9° Cobb at the time of bracing discontinuation, with a mean correction of −11.1°. Curve correction was observed in 13 cases (77 %), stabilization in 3 patients (18 %), and progression in 1 patients (5 %). Among 6 patients who were recontacted at the end of growth, 3 showed a progression of 11,7° Cobb and 1 had undergone surgery.

## Discussion

The present study was undertaken to determine the efficacy of Lyon bracing in a case series of adolescent females with idiopathic thoracic scoliosis. The study was conducted according to the SRS Committee criteria and followed the guidelines on standard of management of idiopathic scoliosis with corrective braces in everyday clinics and in clinical research proposed by the SOSORT [5, 6]. The SRS criteria have been developed to provide uniform criteria for the inclusion of participants in brace studies and the evaluation of results [5]. The SOSORT guidelines provide recommendations concerning the standards of brace management of idiopathic scoliosis, with the aim of ensuring a minimum quality of care, increasing the efficacy of treatment and maximizing the compliance to bracing prescription [6].

Analyses of our case series revealed that the large majority of patients (~85 %) obtained a curve correction after Lyon bracing, whereas a curve stabilization was achieved in ~13 % of cases. Only in one case a curve progression was observed. The greatest correction occurred early during treatment. This may be due to the fact that in the initial phase, bracing acts mostly on the elastic component of the curve, leading to an early, substantial correction. These results can be explained by the capacity of visco-elastic structures to respond promptly to the brace action, with vertebral remodeling occurring later during the course of treatment (in accordance with the law of Hüter-Volkman). As illustrated in Fig. 2, derotation and vertebral remodeling proceed over the entire duration of treatment, assuring further curve correction and its maintenance over time. Results from the present study are consistent with previous reports in JIS and AIS patients treated with Lyon brace, therefore confirming the effectiveness of this bracing device [10–15]. Furthermore, findings from the present study are in agreement with the results of recent studies performed in patients with idiopathic lumbar and thoraco-lumbar curves treated with PASB [16, 17], and indicate that an appropriate conservative approach is successful in most scoliotic curves. In a recent study, Weinstein et al. [18] confirmed that bracing significantly decreased the progression of high-risk curves to the threshold for surgery and that the success rate was higher in patients that had worn the brace for more hours. However, controversies still exist as to whether bracing is truly effective in the management of AIS [19, 20], highlighting the need for high-quality, large-scale clinical trials.

In another recent study conducted following the SRS and SOSORT criteria, Negrini et al. [21] found, in 44 cases, that treatment with several type of braces allowed a curve correction in 86 % of patients with idiopathic thoracic scoliosis and only in the most important cases the Lyon brace was used. A progression was observed in 14 % of cases. These results together with those reported here demonstrate that the adoption of conservative approaches based on the SOSORT and SRS guidelines produce better results than those that followed the SRS criteria only.

A retrospective study conducted in 1,338 AIS patients treated with Lyon brace demonstrated that only the 5 % of curves progressed more than 5° Cobb from the initial magnitude during follow-up [3].

A subgroup analysis in 285 patients with single thoracic curves showed that correction was obtained in 54.26 % cases, stabilization was achieved in 32.25 % of patients, while progression occurred in 12.79 %.

These findings are comparable with previous results from our group, though in our study the success rate was higher with one case of progression (1.5 %) compared to 14 and 12 percent of progression [3, 21].

The same study also reported that, when treatment is started with a Cobb value < 40°, only 2 % of patients eventually require surgery. For Cobb values > 40° at the beginning of treatment, the percentage of patients progressing to surgery is 20 %. These findings indicate that Lyon bracing represents a highly effective conservative approach to AIS, by substantially reducing the need of surgery [13]. Other orthoses are available for the management of idiopathic thoracic curves (e.g., SpineCor, Providence, Milwaukee, etc.) [22–26]. The rate of success of these devices appears to be lower (range 15–60 %) than that achieved by the Lyon brace. It should however be considered that no studies have yet been performed to specifically compare the outcome of treatment with different orthoses in thoracic scoliotic curves, according to the SRS and SOSORT criteria. Furthermore, all the patients included in the study have worn the brace as prescribed. In a previous study in which the results were assessed according to compliance it was determined that curve progression and referral to surgery are lower in patients with high brace compliance. In particular, bracing discontinuation up to 1 month does not impact on the treatment outcome. Conversely, wearing the brace only overnight is associated with a high rate of curve progression [27].

This issue needs to be addressed by future investigations in order to determine the most effective bracing strategy in patients with idiopathic thoracic scoliosis.

About the patients who abandoned the treatment the results showed a progression of curve, at the time of discontinuation, only in the 5 % of cases. Therefore, were not the results to send away the patient but, probably, the trouble of a long term treatment, in particular the failure rate of treatment including the dropouts is 22 % but the surgical rate is lower.

### Limitations of the study

The relatively small sample size of the present work is the main limitation. This is due to SRS criteria that limit the cases but allows comparisons with other studies adopting the same recruitment and evaluation approach. Another limitation of the study is the lack of a control group (i.e., untreated patients), but no ethics committee would allow not to treat structured and progressive scoliosis ranging from 25 to 40° Cobb at 10–12 years of age. Nevertheless, in another our study, it was demonstrated that in all our cases in which the brace is not worn correctly the evolutionary process of scoliosis, confirming its evolutivity, is resumed [27] and recently the efficacy of the brace against the control group was confirmed also in other paper [18].

## Conclusions

In conclusion, our study confirms the efficacy of the Lyon brace in achieving the stabilization and/or correction of the thoracic curves in AIS, due to its biomechanical action on vertebral modeling. Moreover these results together with those reported in a recent literature [16, 17, 21] demonstrate that the adoption of conservative approaches based on the SOSORT and SRS guidelines produce better results than those that followed the SRS criteria only. The SRS and SOSORT criteria for bracing should be considered the methodological and management standards to be followed in future research studies, and will allow meta-analysis to be performed on solid methodological criteria.

## Abbreviations

SRS: Scoliosis Research Society
AIS: Adolescent Idiopathic Scoliosis
JISm: Juvenile Idiopathic Scoliosis
SOSORT: Society on Scoliosis Orthopaedic and Rehabilitation Treatment
LL: Latero-lateral
AP: Anterior-Posterior
